# Intra-abdominal angiosarcoma developing in a capsule of a foreign body: report of a case with associated hemorrhagic diathesis

**DOI:** 10.1186/1477-7819-3-60

**Published:** 2005-09-14

**Authors:** Young-Tae Joo, Chi-Young Jeong, Eun-Jung Jung, Young-Joon Lee, Soon-Chan Hong, Sang-Kyung Choi, Soon-Tae Park, Woo-Song Ha

**Affiliations:** 1 Department of Surgery, Gyeongsang National University Collage of Medicine, Jinju, South Korea

## Abstract

**Backgrounds:**

Angiosarcoma occurs very rarely in the gastrointestinal tract and can present great diagnostic difficulty, especially when it is associated with intraabdominal abscess or granulation tissue.

**Case presentation:**

We report a case where the angiosarcoma was diagnosed after the occurrence of disseminated angiosarcoma and concurrent hemoperitoneum. The tumor developed in the fibrous capsule of a foreign body, which was possibly related to the previous appendectomy twenty years ago, and became a widely disseminated malignant neoplasm in the abdomen. After the operation, the patient's course was dominated by a fatal consumptive coagulapathy. Pathologic examination of the multiple intra-abdominal lesions showed the histological and immunohistological characteristics of the angiosarcoma.

**Conclusion:**

Even though angiosarcoma in the gastrointestinal tract is extremely rare, when dealing with intraabdominal abscess or the gastrointestinal bleeding in patients who have undergone surgery or radiation therapy in the past, the possibility of angiosarcoma should be considered. To make the definite diagnosis of angiosarcoma and to avoid the misdiagnosis of foreign body granuloma, thorough histological examination and immunohistochemical staining may be prerequisite.

## Background

Angiosarcoma is a rare malignant tumor with the incidence of 1%–2% of all sarcomas. It occurs in the skin and subcutis in most cases, and less commonly, it occurs in the liver [1], spleen [2], adrenal gland, and the ovary. Its occurrence in the gastrointestinal tract is extremely rare [3,4], and the development of hemoperitoneum is even more rare in the worldwide [2]. Due to the diagnostic difficulties, intraperitoneal metastases are already present in many patients at the time of diagnosis. For the differential diagnosis, histological findings including immunohistochemistry are most important. We report a case of intraperitoneal disseminated angiosarcoma presenting as hemoperitoneum, which was initially misdiagnosed as intraabdominal abscess.

## Case presentation

**Figure 1:**
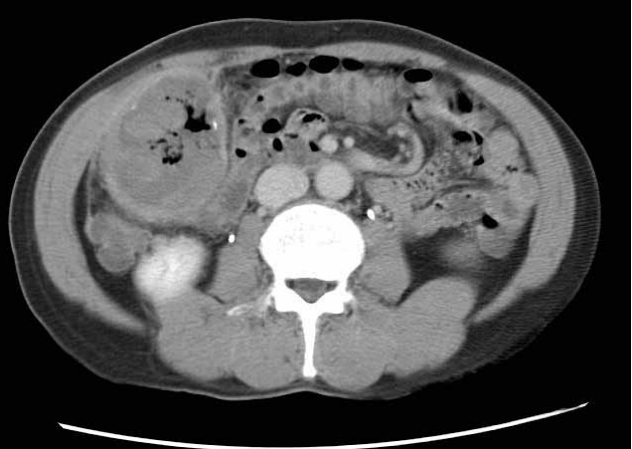
Enhanced abdominal CT scan of upper abdomen showing a 6 cm sized mass in the right lower quadrant of the abdomen at the first operation. The mass contained air bubbles, tiny calcifications and hematoma.

**Figure 2:**
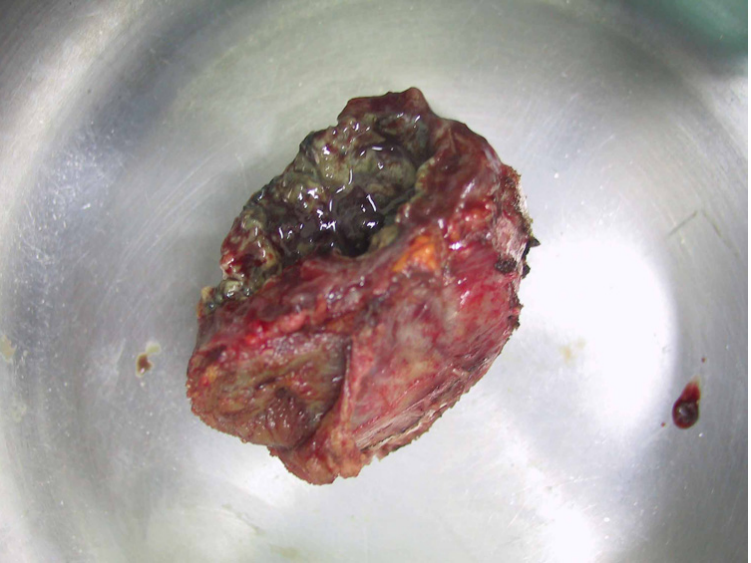
Macroscopic view of the mass in the right paracolic gutter showing a 5 × 3 cm sized, encapsulated mass filled with abscess and granulation tissue.

A 61 year-old male was admitted for constipation and a palpable mass in the periumbilical area. His medical history revealed the surgery for acute perforative appendicitis 20 years ago and occasional intestinal obstruction afterwards. He denied any history of radiation or occupational exposure to chemicals. The small bowel series showed an external compressive lesion in the distal ileum and the computerized tomographic (CT) scan revealed a 6 cm, well-marginated mass in the anteromedial aspect of the ascending colon (Figure 1). Under the suspicion of foreign body granuloma due to gauze (gossypibioma), an exploratory laparotomy was performed. The whole intestine was found to be covered in severe adhesion; however, ascites or blood in the abdominal cavity was absent. In the anteromedial aspect of ascending colon, there was an encapsulated mass 5 cm in size, and it was adhered to ascending colon and distal ileum (Figure 2). The mass was filled with abscess and granulation tissue, and the frozen biopsy reported the diagnosis of abscess. Only resection of the mass was performed, and the patient was discharged without any complications.

Forty days after the discharge, he was admitted again with anemia, abdominal distension, and melana. The colonoscopy revealed the hematoma and the stenosis of the lumen directly above the cecum. The abdominal CT scan showed large volume of blood in the abdomen and multiple peritoneal nodules. The angiography and ^99 m^Tc labeled RBC scan showed active bleeding around the ileocecal valve. Emergent laparotomy was performed and multiple nodules were found on the wall of ileum, liver, mesentery and peritoneum. In the distal ileum, two nodular lesions with 3 cm and 2 cm in size were bleeding actively, and they were adhered to each other. The nodules were diagnosed as sarcoma through the frozen biopsy, and the distal ileum including the pathologic lesion was resected. Seven days later, reoperation was performed due to substantial hemorrhage from the peritoneum, the mesentery, and the small intestine wall. Two days after the last surgery, the patient expired of uncontrollable bleeding due to disseminated intravascular coagulopathy.

**Figure 3:**
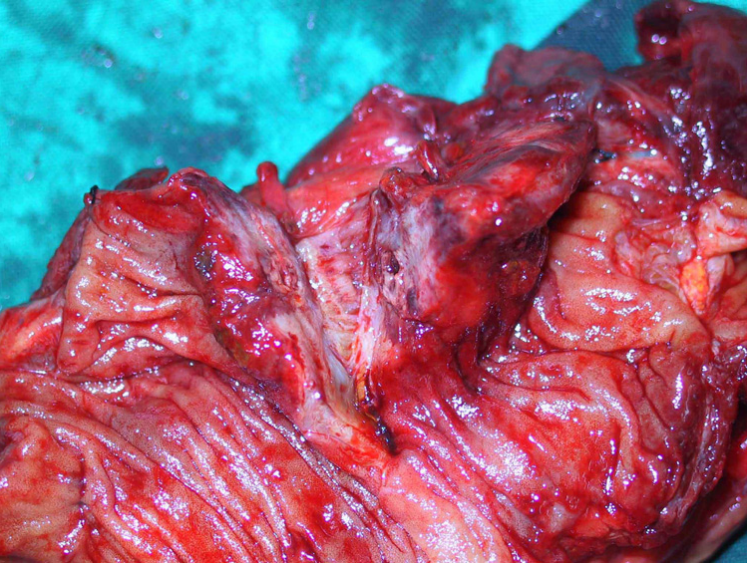
Macroscopic view of the segment of the small intestine showing an ill-defined tan solid mass that involved the entire intestinal layer.

On the macroscopic examination of resected ileum, there were two ill-defined tan solid tumors with mucosal ulceration, each measured 3 × 1.5 cm and 2 cm in diameter, involving the entire intestinal wall and extending to the subserosa of adhered loop. (Figure 3).

**Figure 4:**
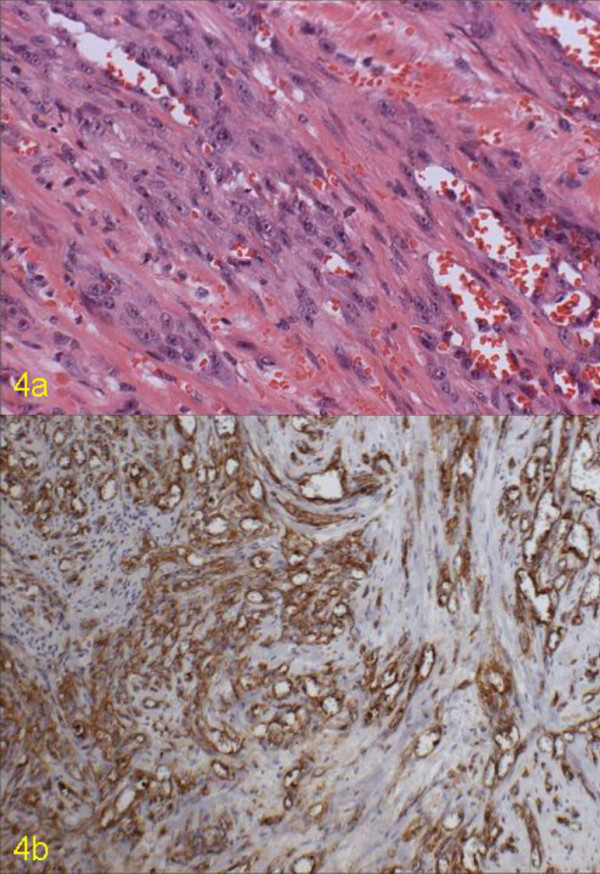
Microscopic findings of the angiosarcoma in the small intestine. 4a) Atypical spindle or epithelioid tumor cells were arranged in sheets, and rudimentary lumen formation was rarely noted. (H&E, × 200) 4b) The tumor cells are strongly positive for anti-CD31. (PAP, × 100)

Under microscope, spindle-shaped or epithelioid cells were arranged as a plate and the rudimentary vessel lumen were detected occasionally (Figure 4a). Separated from these two lesions, several small angiosarcomas containing foreign body granulomas were found in the subserosal layer of the intestine. Also, the nodules of liver and mesentery were diagnosed as metastatic angiosarcomas.

Immunohistochemical staining was performed and tumor cells were positive for CD31, CD34, and vimentin (Figure 4b), whereas negative for factor antigen, CD117, and S-100. The tumor cells were also negative for cytokeratin (AE1/3) and EMA. The foreign body granulomas were surrounded by CD31 positive cells, partially or entirely.

**Figure 5:**
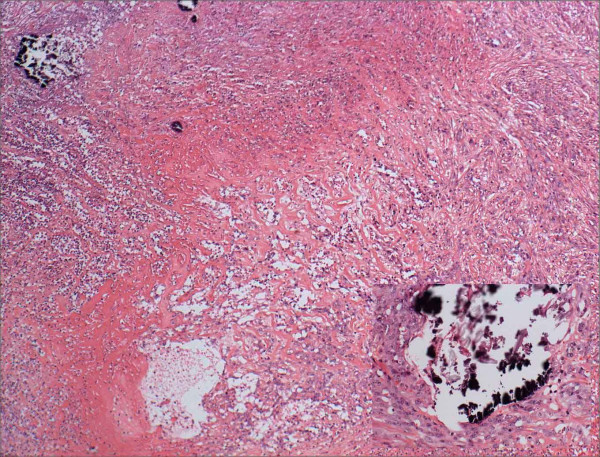
Microscopic finding of the angiosarcoma in the anteromedial aspect of ascending colon. Proliferation of malignant blood vessels was seen in the periphery of the abscess(left upper corner) (H&E, × 40). Inlet: Malignant epitheliod tumor cells were proliferating around the calcified foreign material, which was found in the periphery of the abscess. (H&E, × 200)

The previously resected mass, which was diagnosed as abscess, was reviewed. In a low magnification field, abscess in the center and fibrosis with vascular proliferation in the periphery were noted (Figure 5). However, when the periphery was examined under the high magnification, spindle-shaped or epithelioid cells were arranged as plate patterns, in some area, well-differentiated vessels were formed as similar to the lesions from the ileum. The additional serial sections revealed more foreign materials surrounded by epithelioid tumor cells and the invasion of tumor cells to the blood vessel. The tumor cells were positive for CD31 and CD34, and negative for cytokeratin. This supported the final diagnosis of a foreign body granuloma associated-angiosarcoma.

## Discussion

Although the causality has not been clearly elucidated yet, several factors have been reported to be related to the development of angiosarcoma. Vinyl chloride, arsenic, and thorium dioxide have been reported to cause angiosarcoma in the liver [5]. The radiation and chronic inflammation are important predisposing factors for angiosarcoma [6,7], and rarely, angiosarcoma may develop in association with foreign materials, especially metals, Dacron graft or a retained surgical sponge. According to our review of the literature, 19 cases of angiosarcoma caused by a foreign material have been reported with 4 cases developing in the abdominal cavity. All of these 4 cases developed in relation to a gauze retained for a prolonged period after previous abdominal surgery [8-11]. The formation of fibrotic capsule surrounding the vicinity of foreign body has been reported to be an important factor for developing angiosarcomas [8,9]. In present case, when considering the facts that foreign material was identified in the nodules of ileum and ascending colon, and also in the previously resected tumor, and that there was a close topograghic association of the previous appendectomy site with the developing site of the angiosarcoma, it can be speculated that the foreign material from the previous appendectomy was the cause of foreign body associated angiosarcoma, whether the foreign material was gauze or suture material.

Gastrointestinal angiosarcoma usually presents with abdominal pain, bleeding, and obstruction. Especially in cases of small bleeding angiosarcoma in the small intestine, the angiography or ^99 m^T_c_-labelled RBC scan may be needed to identify the bleeding focus [12]. In present case, because of the invasion of tumor cells through the serosa, hemorrhage into the abdominal cavity and local metastasis may have developed more readily rather than the gastrointestinal bleeding.

For the definite diagnosis, histological findings are most important and immunohistochemical staining is necessary for the differential diagnosis from undifferentiated malignant tumor, malignant melanoma, and smooth muscle sarcoma [13]. The cells of angiosarcoma are positively stained for vimentin, the endothelial cell markers such as CD31, CD34, and factor, and negatively for epithelial marker such as cytokeratin and EMA, however, in the epithelioid angiosarcoma, it may be stained as positive [14].

Many reported various staining findings in angiosarcoma, and such diverse staining findings imply the diversity for the degree of differentiation seen in angiosarcoma and the variation for the expression of the markers [13,14].

## Conclusion

Angiosarcoma in the gastrointestinal tract is extremely rare, and it can present with diverse symptoms. If the intraabdominal abscess or the gastrointestinal bleeding is detected in patients who have undergone surgery or radiation therapy in the past, the possibility of angiosarcoma should be considered. To make the definite diagnosis of angiosarcoma and to avoid the misdiagnosis of foreign body granuloma, thorough histological examination and immunohistochemical staining may be prerequisite.

## Competing interests

The author(s) declare that they have no competing interests.

## Authors' contributions

YTJ participated in the serial operations of the patient, prepared the manuscript for submission and resubmission

CYJ participated in the serial operations of the patient, prepared the manuscript for submission and resubmission

EJ participated in the serial operations of the patient, prepared the manuscript for submission and resubmission

YJL participated in the serial operations of the patient, prepared the manuscript for submission and resubmission

SCH participated in the serial operations of the patient, prepared the manuscript for submission and resubmission

SKC, participated in the serial operations of the patient, prepared the manuscript for submission and resubmission

WH participated in the serial operations of the patient, prepared the manuscript for submission and resubmission

STP participated in the serial operations of the patient, prepared the manuscript for submission and resubmission, and helped in the final editing process.

All authors approved the final version.
